# Studying host genetic background effects on multimorbidity of intestinal cancer development, type 2 diabetes and obesity in response to oral bacterial infection and high‐fat diet using the collaborative cross (CC) lines

**DOI:** 10.1002/ame2.12151

**Published:** 2021-02-14

**Authors:** Asal Milhem, Hanifa J. Abu Toamih‐Atamni, Luna Karkar, Yael Houri‐Haddad, Fuad A. Iraqi

**Affiliations:** ^1^ Department of Clinical Microbiology and Immunology Sackler Faculty of Medicine Tel‐Aviv University Tel Aviv Israel; ^2^ Department of Prosthodontics Dental School The Hebrew University Hadassah Jerusalem Israel

**Keywords:** high‐fat diet (42% fat), intestinal cancer, multimorbidity, obesity, oral bacterial infection, type 2 diabetes (T2D)

## Abstract

**Background:**

Multimorbidity of intestinal cancer (IC), type 2 diabetes (T2D) and obesity is a complex set of diseases, affected by environmental and genetic risk factors. High‐fat diet (HFD) and oral bacterial infection play important roles in the etiology of these diseases through inflammation and various biological mechanisms.

**Methods:**

To study the complexity of this multimorbidity, we used the collaborative cross (CC) mouse genetics reference population. We aimed to study the multimorbidity of IC, T2D, and obesity using CC lines, measuring their responses to HFD and oral bacterial infection. The study used 63 mice of both sexes generated from two CC lines (IL557 and IL711). For 12 weeks, experimental mice were maintained on specific dietary regimes combined with co‐infection with oral bacteria *Porphyromonas gingivalis* and *Fusobacterium nucleatum*, while control groups were not infected. Body weight (BW) and results of a intraperitoneal glucose tolerance test (IPGTT) were recorded at the end of 12 weeks, after which length and size of the intestines were assessed for polyp counts.

**Results:**

Polyp counts ranged between 2 and 10 per CC line. The combination of HFD and infection significantly reduced (*P* < .01) the colon polyp size of IL557 females to 2.5 cm^2^, compared to the other groups. Comparing BW gain, IL557 males on HFD gained 18 g, while the females gained 10 g under the same conditions and showed the highest area under curve (AUC) values of 40 000‐45 000 (min mg/dL) in the IPGTT.

**Conclusion:**

The results show that mice from different genetic backgrounds respond differently to a high fat diet and oral infection in terms of polyp development and glucose tolerance, and this effect is gender related.

## 1 INTRODUCTION

Multimorbidity is the existence of multiple chronic conditions and diseases associated with an elevated risk of death, disability, low quality of life, and environmental and genetic factors. The term intestinal cancer refers to a slowly developing cancer that begins as a tumor or tissue growth on the inner lining of different parts of the intestines.^1^ Epidemiological and molecular evidence links obesity and metabolic status with inflammation and an increased risk of many cancers.^2^ There are several risk factors involved in intestinal cancer development, with one of the most important factors being age. Epidemiological studies show that the majority of cases are diagnosed among individuals aged 50 years old and older. A sedentary lifestyle characterized by a lack of physical activity, along with a fat and carbohydrate rich (western) diet, high alcohol intake, and long‐term smoking are also considered as leading risk factors for colorectal cancer (CRC) development.^3^

A high‐fat diet (HFD) plays an important role in the disease risk for type 2 diabetes (T2D), obesity, and intestinal cancer through inflammation and other obscure biological mechanisms. Obesity and related metabolic disturbances are also closely associated with pathologies that represent a significant burden on global health. T2D leads to a hyperinflammatory response, which disrupts the balance of oral microbiota, subsequently resulting in inflammation, which accelerates intestinal cancer development.^4^ Finally, studies of familial heritability and the genome‐wide associations study (GWAS) have confirmed a significant role of genetic factors in colon cancer development.^5^ Another study has shown that oral bacteria including *Porphyromonas gingivalis* (Pg) and *Fusobacterium nucleatum* (Fn) may colonize the host gut, which subsequently develops local inflammation that may trigger/initiate intestinal cancer development.^6^, ^7^, ^8^ In addition, a previous study by Demmer et al reported that oral bacteria were related to inflammation and insulin resistance among diabetes‐free adults.^9^

Our recent study showed that different strains of mice respond differently to dietary and infection challenge‐induced co‐morbidity of T2D and obesity.^10^ This variation is explained to a significant extent by genetic variability. A better understanding of how this genetic variation translates into different clinical manifestations will eventually highlight pathways through which dietary composition may initiate or accelerate inflammatory disease processes and indicate mechanisms through which disease can potentially be prevented.

Recently, it was demonstrated that genetically highly diverse sets of recombinant inbred mouse lines (RIL), collectively named the Collaborative Cross (CC), can be used as a tool for the identification of risk genes in complex human disease.^8^ The CC was developed as the next generation of mouse genetic reference population, which allows time‐ and cost‐efficient mapping of quantitative trait loci (QTLs) associated with complex traits.^11^, ^12^, ^13^, ^14^, ^15^, ^16^, ^17^, ^18^, ^19^, ^20^ The CC population is a large panel of recombinant inbred (RI) strains derived from a genetically diverse set of eight founder strains: A/J, C57BL/6J, 129S1/SvImJ, NOD/LtJ, NZO/HiLtJ, CAST/Ei, PWK/PhJ, and WSB/EiJ. The key features of the CC genetic reference population with gene mapping are that a very large number of variants segregate in the population (there are over 36 million SNPs)^12^ and the relatively high level of recombination present compared to other mouse RI sets.

Finally, our previous and current results support the use of the CC mouse genetic reference population as a unique and powerful platform for studying the multimorbidity of intestinal cancer, T2D, and obesity in response to a high‐fat diet and oral bacterial co‐infection, as a step towards identifying disease‐related genetic factors.

## 2 METHODS

### 2.1 Ethical statement

All the experiments and mouse usage described in this study were compatible with the standards for care and use of laboratory animals and approved by the Institutional Animal Care and Use Committee (IACUC) of Tel Aviv University (TAU), Israel (IACUC no. 01‐19‐013).

### 2.2 Study cohort

63 mice were used in this study from two different CC lines, IL711 and IL557, which have different genetic backgrounds; both sexes were represented in each study group.^6^, ^12^, ^14^, ^17^, ^18^, ^21^ High molecular weight genomic DNA for the CC lines was initially genotyped using the mouse diversity array (MDA), which consists of 620 000 SNPs, and re‐genotyped by mouse universal genotype array (MUGA‐7500 markers) and eventually with MegaMuga (77 800 markers) SNP arrays to confirm their genotype status and variations in their genetic structure.^12^ These mice were maintained at our animal facility at TAU under suitable and agreed ethical conditions of temperature (21‐23°C), humidity and daily supervision. Mice were weaned at the age of 3 weeks old, and then maintained separately by line and sex, with a maximum of five mice in an open‐top cage and free access to water and rodent chow diet. Summary of the used mice in this study and their assignments in the different experimental groups are presented in (Table 1).

**TABLE 1:** Summary table of the total number (N) of mice from each CC line (IL557 and IL711), males and females separately, assessed in each study group

CC Line	HFD (42% fat)				CHD (11% fat)				Total
	Inf (+)		Inf (−)		Inf (+)		Inf (−)		
	♀	♂	♀	♂	♀	♂	♀	♂	
IL557	2	3	2	5	3	2	5	3	25
IL711	5	4	5	5	5	5	5	4	38
Total	7	7	7	10	8	7	10	7	63

### 2.3 Study design

The experiments started when mice were 8 weeks old and lasted for a period of 12 weeks, with the mice on either a high‐fat dietary (HFD) challenge, with or without bacterial oral infection, or a standard chow diet (CHD), with or without bacterial oral infection. Body weight (g) was determined bi‐weekly, and glucose tolerance ability was determined at the end time point of the experiment. Thus the experiment included four experimental groups as detailed and named below.

*CHD/no‐infection*: This group was maintained on CHD for 12 weeks as a control group for the HFD group and treated with a placebo infection challenge (no bacterial infection). *HFD/no‐infection*: This group was maintained on HFD for 12 weeks and treated with a placebo infection challenge as a control group for the bacterial infection challenge. *CHD/with‐infection*: This group was maintained on CHD for 12 weeks and treated with a bacterial infection challenge as an experimental group for infection effect. *HFD/with‐infection*: This group was maintained on HFD for 12 weeks and treated with bacterial infection challenge as an experimental group for the combined challenges of infection and HFD.

**FIGURE 1:**
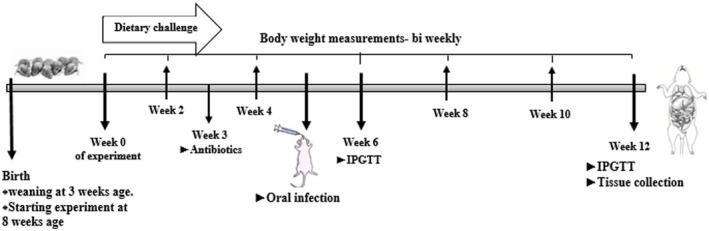
Study design scheme showing timescale and experimental procedures. The experiments started when mice were 8 wk old (start point) and continuing until they were 20 wk old, ie 12 wk. At the start time point, BW was recorded and mice were divided into two dietary groups: HFD (*42% fat) or CHD (*11% fat, control group). At week 5 of the experiment (13 wk old), infection was performed perorally with mixed‐oral bacteria for the experimental groups and placebo‐infection without bacteria for control groups. At week 12 of the experiment, glucose tolerance ability was assessed by IPGTT, the mice were sacrificed and tissues were collected for further study including small and large intestines. CHD, chow diet; BW, body weight; HFD, high‐fat diet; IPGTT, intraperitoneal glucose tolerance test. *%, Kcal/Kg from fat (metabolized energy)

Figure 1 shows the study design scheme, including the time scale and the study groups for the experiment procedures. The study ran from when the mice were 8 weeks old (start point) until they were 20 weeks old, ie a 12 week period.

### 2.4 Dietary challenge

Mice were weaned at the age of 3 weeks and maintained until 8 weeks of age on a standard rodent chow diet (CHD; Altromin 1324 IRR, Altromin Spezialfutter GmbH & Co., Germany), which provides 11% Kcal from fat, 24% from protein, and 65% from carbohydrates. The dietary challenge started when the mice were 8 weeks old, when they were maintained on either CHD or HFD for the 12‐week period of the experiment. The high‐fat diet (HFD; TD.88137) was considered equivalent to a western diet, and was supplied by Teklad Global (Harlan Inc, Madison, WI, USA). The HFD provided 42.0% Kcal from fat, 15.3% from protein, and 42.7% from carbohydrates.

### 2.5 Bacterial culture

*Porphyromonas gingivalis* (Pg) strain ATCC 33277 and *Fusobacterium nucleatum* (Fn) strain PK 1594 were grown in peptone yeast extract containing hemin and vitamin K (Wilkins Chalgren Broth, Oxoid Ltd, Basingstoke, UK), in an anaerobic chamber with 85% N_2_, 5% H_2_, and 10% CO_2_, followed by 3 washes of two minutes, each, in phosphate‐buffered saline (PBS). The bacterial concentration was measured spectrophotometrically and standardized to OD_650_nm = 0.1 for Pg, corresponding to 10^10^ bacteria/ml; and OD_660_nm = 0.26 for Fn, corresponding to 10^9^ bacteria/ml. Before the infection challenge, the two strains of bacteria were mixed together in a 1:1 (Pg:Fn) ratio.

### 2.6 Oral infection challenge

Before the infection challenge, mice were treated with antibiotics to standardize the oral microbiota status of the different mice, using sulfamethoxazole (10/500 mL) water administered for 10 days, followed by 3 days’ recovery (antibiotic‐free). The mice were then orally infected with 400 μL per mouse of the mixed oral bacteria (Pg and Fn). The infection procedure was repeated 3 times, on days 1, 3 and 5 of week 5. In parallel, control groups of the placebo infection were treated with 400 µL of 2% CMC in distilled water and 1% PBS (CMC:PBS ratio of 2:1).

### 2.7 Body weight measurements

During the 12 weeks of the experiment, the body weight (BW) of the mice was recorded bi‐weekly using an electronic scale with 0.1 g accuracy.

### 2.8 Intraperitoneal glucose tolerance test (IPGTT)

The IPGTT was performed at the end of the experiment to detect disturbances in glucose tolerance ability as an indicator of the development of T2D. On the morning of the IPGTT, the mice were fasted for 6 hours (6:00‐12:00 am), with free access to water. Fasting blood glucose levels (time 0) were then determined from tail blood, and subsequently a solution of glucose (2.5 mg glucose per g mouse body mass) was administered by intraperitoneal (IP) injection. Thereafter, blood glucose levels were monitored at different time points during the following 180 minutes (15, 30, 60, 120, and 180 minutes after glucose injection). The mice were then returned to their cages with free access to food and water for overnight recovery.

### 2.9 The area under the curve (AUC)

IPGTT results were corrrelated to glucose tolerance ability by calculating the AUC of the 180‐minute glucose tolerance clearance process. Calculation of AUC was conducted according to the trapezoid rule from time 0 to 180 min after glucose injection to quantitatively evaluate glucose clearance activity. AUC between any two time points was calculated as (time difference in minutes between sequential reads) × (glucose level at 1st time point + glucose level at 2nd time point)/2). The total AUC value of the 180‐minute IPGTT was calculated as the sum of the AUC between each set of two time points, total AUC0‐180 = AUC0‐15 + AUC15‐30 + AUC30‐60 + AUC60‐120 + AUC120‐180.

#### 2.9.1 Intestines collection

At the end point of the experiment (at 20 weeks old), the mice were sacrificed by CO_2_ inhalation and the intestines collected.

### 2.10 Intestinal preparation for polyp counts

Intestines were collected and soaked in a small plate filled with phosphate buffered saline (PBS) until wash (5‐10 minutes), which makes the tissue easier to use. Small and large intestines were then washed and cleaned at least twice with PBS. The small intestines were divided into three equal segments; proximal, middle, and distal. Each segment including the colon was horizontally opened/cut by razor and spread over 154 cm^2^ Whatman paper and fixed overnight in 10% neutral buffered formalin (NBF). The length and width of each was measured by ruler.

The intestines were washed with 70% ethanol and then stained with 0.02% methylene blue (1.5 minutes for each paper) and kept overnight in PBS on the shaker. The number of polyps, length in cm, and size (width × length) in cm^2^ of each segment of intestines were recorded.

### 2.11 Data analysis

Data analysis was performed using the statistical software package SPSS version 25 (IBM SPSS Statistics 25). Analysis of variance (one‐way ANOVA) was performed to test the differences in body weight (BW), glucose tolerance, intestinal weight, intestinal length, intestinal size, length, size, and total polyp count between the CC lines. All presented data are expressed as means ± standard error. Examination of the data (not shown) confirmed that the data sets meet the requirements for normality and equal variance for using an ANOVA test.

## 3 RESULTS

Herein, we present a data analysis of 63 mice generated from two genetically different CC lines, with both sexes represented. These mice were exposed to a dietary challenge (HFD vs. CHD) with or without oral co‐infection challenges during a 12‐week experimental period. The results display the response of the mice to the challenges, and the variation within and between the CC lines and both sexes, from the four different experimental groups. The response variations in the number of polyps in male and female mice after 12 weeks on either HFD or CHD under infection or non‐infection conditions were determined, as were the length and size of intestine measurements, which were used in data analysis.

### 3.1 Polyp numbers vary between the CC lines in response to dietary and infection challenge

**FIGURE 2:**
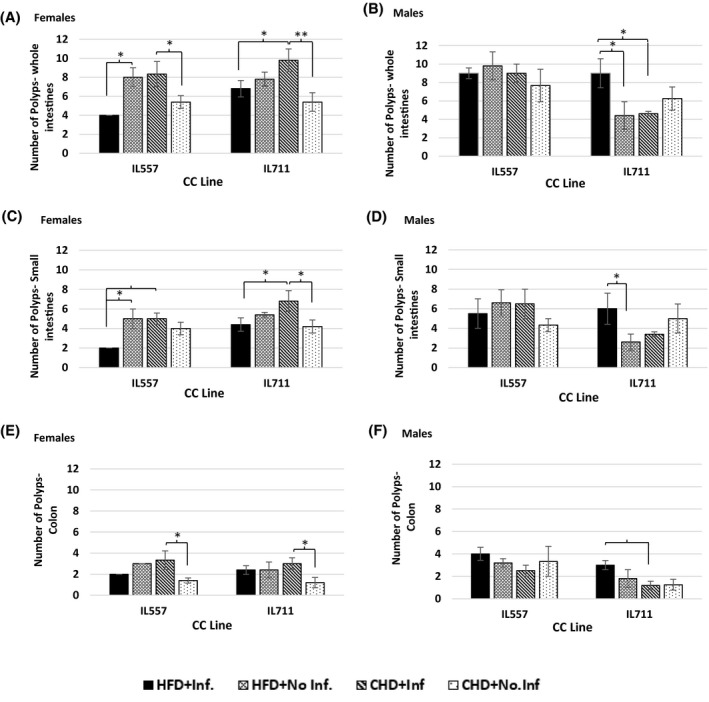
Number of polyps following 12 wk of standard chow diet (CHD) versus high‐fat (42% fat) diet (HFD) challenge and infection challenge in male and female populations. X‐axis, CC lines; Y‐axis, the number of polyps in whole intestines (A and B), small intestines (C and D) and in colon (E and F). * and **indicate significant *P* values of <.05 and <.01, respectively

Our results show that there is a variation in polyp number between the different CC lines on HFD and CHD. To evaluate the effect of the HFD on the number of polyps developed, we compared the control group (CHD/no‐infection) with the non‐infected group on HFD (HFD/no‐infection). Females from both IL557 and IL711 in the control groups developed the same number of polyps throughout the intestines (small and large intestines), averaging 5.40 ± 0.829 polyps, while female mice on HFD developed more polyps, averaging 8 ± 1 polyps in IL557 and 7.8 ± 0.73 polyps in IL711 (Figure 2A). In both small and large intestines, separately, female mice on HFD developed more polyps compared to control mice, but the numbers were not significantly different, averaging 5 ± 1 polyps in IL557 and 5.4 ± 0.244 polyps in IL711 in the small intestines, and 3 ± 0.00 and 2.40 ± 0.40 in the colon of IL557 and IL711, respectively (Figure 2C,E). Interestingly, in the male population, the different lines responded differently to the dietary challenge as shown in Figure 2B,D,F. Across the whole of the intestines, IL557 on HFD developed more polyps, averaging 9.8 ± 1.53, compared to the control mice group with an average of 7.67 ± 1.76 polyps, while IL711 on HFD developed fewer polyps compared to control mice. The same pattern was also seen in the small intestines but the opposite pattern was observed in the colon, where IL557 developed in average 3 ± 0.5 polyps in both groups while IL711 on HFD developed more polyps (1.5 ± 0.5) compared to the control group, with 1 ± 0.5 polyps on average.

The effect of the infection on polyp development, along the whole intestines was highly significant in the female population. Infected IL711 mice developed on average 9.8 ± 1.2 polyps, significantly more than the control group with an average of 5.4 ± 0.98 polyps (*P* < .01), and more than IL557. The infected mice of IL557 displayed the same pattern, by developing 3 more polyps compared to control groups (Figure 2A). In the colon, the infected mice developed a higher number of average polyps, 3.3 ± 0.0, compared to control groups within a line and between the lines (Figure 2C,E). In the male population, there were no significant differences between the infected mice and control groups (Figure 2B,D,F).

The combination of HFD and infection significantly affected (*P* < .05) the whole intestines and the small intestine in the female population compared to other groups (Figure 2A). In the small intestines, both lines developed significantly (*P* < .05) fewer polyps compared to infected mice on CHD, with an average of 3 and 2.4 less in IL557 and IL711, respectively. In the colon, however, no significant differences were observed between the infected and control groups (Figure 2E). Infected mice on HFD from the IL711 male population responded completely differently by developing a higher number of polyps compared to other groups from the same line. In the small intestines there were significant differences, with an average of 3.4 more polyps than in non‐infected mice on HFD (*P* < .01) (Figure 2). IL711 mice showed the same pattern in the colon, with significantly more polyps compared to infected mice on CHD. In addition, infected mice from IL557 on HFD developed a higher number of polyps compared to other groups, averaging 4 ± 0.57, while in the small intestines of the same mice, developed fewer polyps, which was not significantly different from control values (Figure 2D,F).

### 3.2 The effect of the dietary and infection challenges on the length of the intestines varies between IL577 and IL711

**FIGURE 3:**
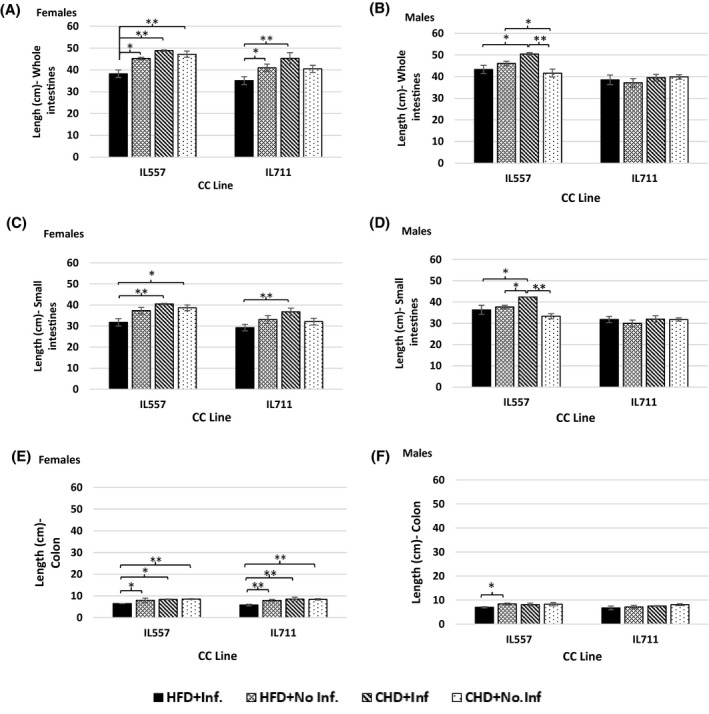
Length of intestines following 12 wk of standard chow diet (CHD) versus high‐fat (42% fat) diet (HFD) challenge and infection challenge in male and female populations. X‐axis, CC lines; Y‐axis, length (cm) of whole intestines (A and B), small intestines (C and D) and colon (E and F). * and **indicate significant *P* values of <.05 and <.01, respectively

In the female population, the same pattern of changes was observed in both lines (Figure 3A,C,E). In IL557 the infected mice had the longest intestines, with an average of 48.8 ± 0.40 cm of whole intestines compared to other groups, but this value was close to the values for the control group, with an average of 47.2 ± 1.42 cm, and control mice on HFD, with average 45.25 ± 0.55 cm. Interestingly, the combination of HFD and infection resulted in a significant shortening (*P* < .05) of the intestines compared to other groups, especially in the length of the colon, which in IL557 and IL711 averaged 6.5 ± 0.0 cm and 5.84 ± 0.42 cm, respectively, significantly different (*P* < .05 and *P* < .01, respectively) from other groups. In the male population, the whole intestines of infected IL557 mice on CHD averaged 50.5 ± 0.6 cm, significantly different from the control group (CHD/no‐infection) (*P* < .01), and from infected mice on HFD (*P* < .05) (Figure 3B). The length of the small intestines was, significantly different, in control mice compared to non‐infected mice on HFD (*P* < .05), and also significantly different in infected mice from other groups (Figure 3D), while the length of the colon was close to that of other groups (Figure 3F). In IL557, there was a significant difference (*P* < .05) in the length of the colon between infected and non‐infected mice on HFD, with an average of 1.42 cm.

### 3.3 Significant variations in the size of intestines between IL557 and IL711

**FIGURE 4:**
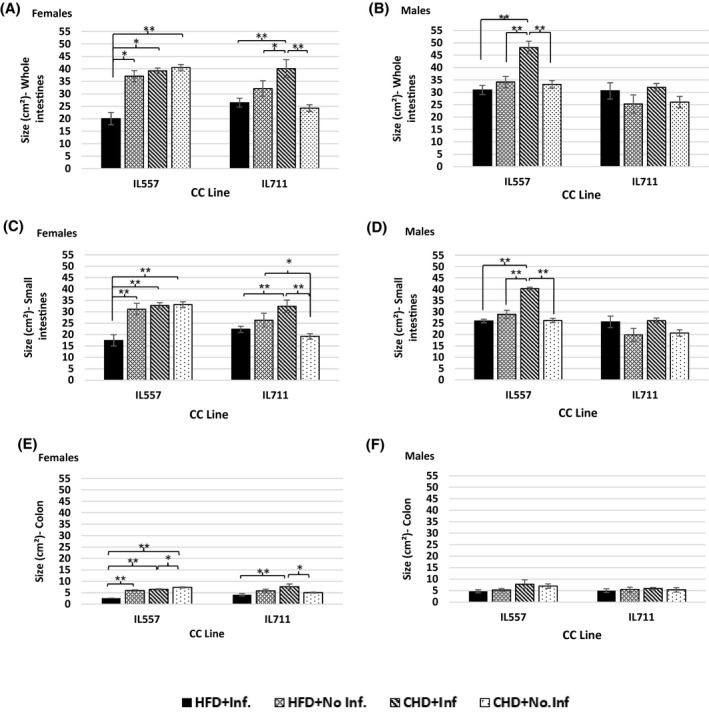
Size of intestines following 12 wk of standard chow diet (CHD) versus high‐fat (42% fat) diet (HFD) challenge and infection challenge in male and female populations. X‐axis, CC lines; Y‐axis, the size (cm^2^) of whole intestines (A and B), small intestines (C and D) and colon (E and F). * and **indicate significant *P* values of <.05 and <.01, respectively

The IL711 male population did not show any significant variations in the size of intestines in response to HFD and an infection challenge. In contrast, the size of the intestines, and in particular the small intestines, of infected male IL557 mice on CHD was significant larger (*P* < .01) than other groups and challenges (Figure 4B), with an average size of 48.09 ± 2.5 cm^2^ for the whole intestines, and an average of 40.2 ± 0.66 cm^2^ for the small intestines (Figure 4D). In the female population, the infected IL711 mice maintained on CHD presented the largest size of whole intestines, which was significantly different (*P* < .01) from other groups (Figure 4A). The females in the control group presented the smallest intestines, with an average for the small intestines of 19.22 ± 1.2 cm^2^ compared to infected mice (*P* < .01) and non‐infected mice on HFD (*P* < .05). Control female IL557 mice had almost the same size of small intestines as infected mice and non‐infected mice on HFD (Figure 4C), except for the colon, which in the control mice group was significantly (*P* < .05) larger than in infected mice, with an average of 7.35 ± 0.22 cm^2^. Interestingly, in IL557 the combination between HFD and infection reduced the size of intestines significantly (*P* < .01) in both small (17.45 ± 2.4 cm^2^) and large intestines (2.6 ± 0.35 cm^2^) compared to all the other groups (Figure 4C,E).

### 3.4 Difference in the effect of HFD on glucose tolerance between IL557 and IL711 mice, with a strong sex effect

**FIGURE 5:**
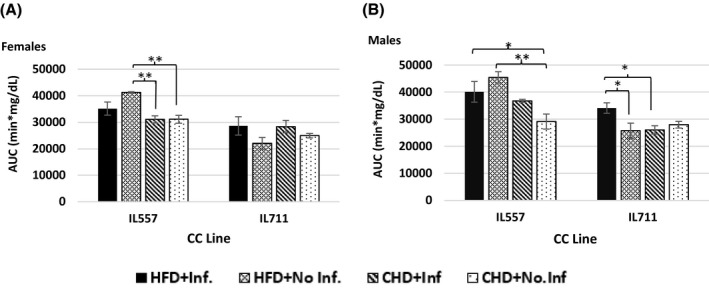
Blood glucose tolerance presented as area under curve (AUC, min mg/dL) of 180 min of glucose clearance test in male and female populations. Y‐axis, AUC in females (A) and males (B); X‐axis, different CC lines following 12 wk of CHD and HFD (42% fat) challenge, with or without infection challenge. * and **indicate significant *P* values of <.05 and <.01, respectively

IL557 mice on HFD revealed, significantly higher values of AUC compared to the control group (*P* < .01) in both male and female populations, with average values of 45 432 ± 2088.31 min mg/dL and 451 250 ± 270.00 min mg/dL, respectively. The combination of HFD and infection significantly affected (*P* < .05) the male population of IL557 compared to the control group, with a difference of 10987.5 min mg/dL. Interestingly, IL711 females had lower AUC values in response to HFD consumption, but with no significant differences, while in the male population the combination of HFD and infection significantly increased (*P* < .05) the differences from the non‐infected group on HFD and the infected group on CHD, with average differences of 24 113.75 ± 1932.86 min mg/dL, respectively (Figure 5A,B).

### 3.5 Male and female IL557 mice gained more weight than IL711 mice under the same conditions of diet and infection

**FIGURE 6:**
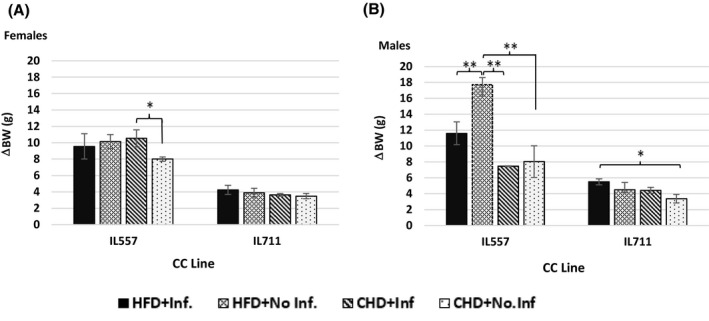
Change in body weight (g) following 12 wk of HFD versus CHD dietary challenge and infection challenge for females (A) and males (B) of the different CC lines. X‐axis, two different CC lines; Y‐axis, body weight gain (g). Change in body weight (ΔBW) was calculated by subtracting the body weight at the initial time point (week 0) from that at the end time point (week 12), ie ΔBW = BW12 − BW0. * and **indicate significant *P* values of <.05 and <.01, respectively

In our previous studies, it was shown that line and sex have major effects on body weight gain in response to dietary and infection challenge.^12^, ^13^, ^22^ In this study, while in IL711 females there were no significant changes in body weight gain between the different groups, body weights of infected IL711 males on HFD were significantly higher than in the control group (*P* < .05), with an average weight gain of 5.5 ± 0.37 g. IL557 males were susceptible to HFD consumption, and they gained on average 17.72 ± 0.88 g in weight, significantly more (*P* < .01) than the other three groups (Figure 6B), while infected IL557 females on CHD reached an average weight gain of 1053 ± 1.03 g, significantly higher (*P* < .05) than control groups.

### 3.6 Heatmap of multimorbidity of polyp counts, AUC, BW, and length and size changes in the development of intestines in IL557 and IL711 mice

**FIGURE 7:**
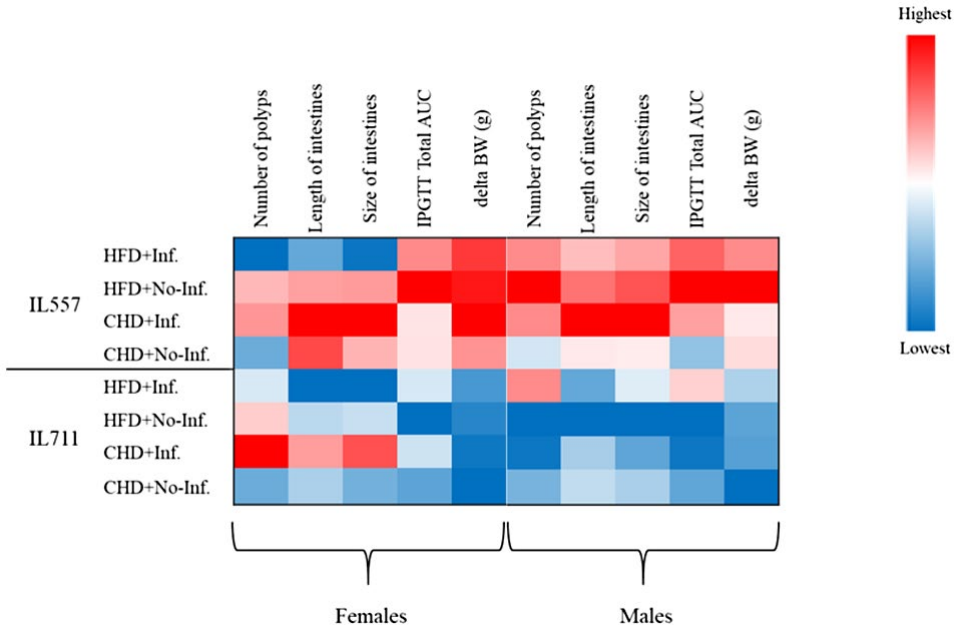
Heatmap of polyp counts, glucose tolerance, body weight gain, and length and size of intestines at week 12 in different two CC lines (IL557 and IL711) in response to a 42% high‐fat diet (HFD) or chow diet (CHD), and with (+Inf.) or without (No‐Inf.) oral bacterial infection challenges in female and male populations. Blue and red colors indicate low and high scores, respectively, for the studied traits

To be better visualize and understand the phenotype patterns of the assessed traits, including polyp counts, diabetes, obesity, length and size changes of the intestines – in the CC lines studied, and between the sexes, as affected by the high fat (HFD) or chow diets (CHD), with or without oral bacterial infection, we have developed a heatmap of these traits and searched for an association in development and severity between these traits. As presented in Figure 7, overall, IL711 mice showed low values (blue) for the assessed traits compared with IL557 mice, which indicates, to be more resistant to the effects of the dietary and infection challenges on the development of these traits. IL557 mice showed high values (red) compared to IL711 mice for the assessed traits when maintained on HFD with infection, HFD without infection and CHD with infection, but not when given chow diet only. Furthermore, IL557 males and females had a significant response to HFD, especially with AUC measurements and BW changes. IL557 males of were more sensitive to the combination of HFD and infection as shown by the higher values for BW, AUC, polyp counts and intestines measurements in males than females. The infection challenge affected the intestinal traits of females from both lines, and IL557 males on CHD, while IL711 males of were resistance to the challenge (Figure 7).

**FIGURE 8:**
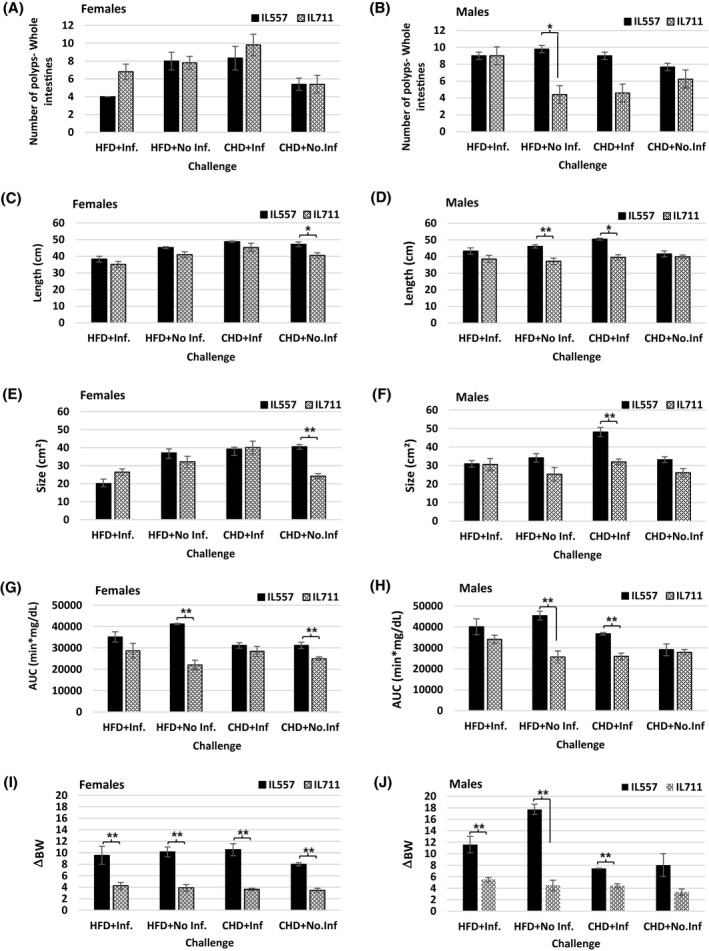
Comparisons of polyp counts, glucose tolerance, body weight gain, and length and size of intestines at week 12 in male and female mice from the IL557 and IL711 CC lines in response to a 42% high‐fat diet (HFD) or chow diet (CHD), and with (+Inf.) or without (No‐Inf.) oral bacterial infection challenges in female and male populations. Number of recorded polyps in the whole intestines in each experimental group (HFD + Inf., HFD + No‐Inf., CHD + Inf. and CHD + No‐Inf.) in female and male mice from IL557 and IL711 CC lines are presented in (A) and (B), respectively, while length of the intestines in the same groups in female and male mice are presented in (C) and (D), respectively. (E) and (F) present intestines sizes for females and males, respectively, in these two lines. AUC values in female and male mice in these studied groups are presented in (G) and (H), respectively, and body weight gain traits (ΔBW) for female and male mice are presented in (I) and (J), respectively. * and **indicate significant *P* values of <.05 and <.01, respectively

Finally, we present in Figure 8 comparisons of polyp counts along the whole intestines, glucose tolerance as determined by AUC values, body weight gain, and length and size of intestines traits at week 12 in male and female mice of both IL557 and IL711 CC lines in response to a high‐fat (42%) diet (HFD) or a chow diet (CHD), and with (+Inf.) or without (No‐Inf.) oral bacterial infection challenges in female and male populations. This figure further analyzes the data presented in the heatmap in Figure 7, and shows which traits were found to be non‐significant or significant at levels *P* < .05 and *P* < .01 in female and male mice of the studied lines in the different experimental groups.

The results presented here, show that body weight gain was highly significantly (*P* < .01) increased in female IL557 mice compared with IL711 females in all experimental groups, while in male mice body weight gain was highly significant (*P* < .01) in all groups except the CHD + No‐Inf. group. The same significance level (*P* < .01) was also observed in AUC values between females in the HFD + No‐Inf. and CHD + No‐Inf. group, and males in HFD + No‐Inf. and in CHD + Inf groups. Intestine size was highly significantly different (*P* < .01) between IL557 and IL711 females in the CHD + No‐Inf. group only, and between the male mice in the CHD + Inf group. In the case of the length of the intestines, a significant variation (*P* < .05) was observed between IL557 and IL711 females in the CHD + No‐Inf. group only, and between male mice in two experimental groups, HFD + No‐Inf. and CHD + Inf. Finally, the number of polyps was found to be a significant variant (*P* < .05) between the two lines only in HFD + No‐Inf. Group, with no significant defference between sexes.

## 4 DISCUSSION

A previous study by our group, using the CC mouse model population, confirmed that different CC lines respond differently to HFD, with males and females of the different lines varying significantly in T2D development and progression in response to two diet challenges, CHD and HFD.^8^, ^22^, ^23^, ^24^ Furthermore, a recent study from our lab has reported comorbidity between T2D and obesity.^10^

This study also showed that the response of the mice to the interaction between infection and the diet challenges varied between the lines and between sexes of the same lines.^10^ These important findings demonstrate the power of CC mice for studying the impact of genetic background on multimorbidity of intestinal cancer, obesity, and T2D development.

Diet and oral infection are two of the main factors that cause chronic inflammation, which has been verified as the most important preventable cause of cancer and other chronic diseases including T2D. Many previous studies have provided evidence that oral microorganisms are associated with gastrointestinal cancers, and evidence suggests that specific bacterial infections promote the development of certain diseases.^25^ *Fusobacteria* bacteria, which are found in human normal oral flora, have been shown to causes excessive immune responses and may activate cancer growth genes, and *Porphyromonas gingivalis* bacteria, also found in human normal oral flora, appear to promote distant metastasis and chemoresistance to anti‐cancer agents.^26^ In regard to the development of chronic systemic inflammation, HFD‐related inflammation leads to the failure of adipocytes to effectively remove circulating free fatty acids and is pivotal to disease progression and the development of complications, including T2D, CVD, liver disease, and certain types of cancers during shifts and alterations in the gut microbiota.^27^ Accordingly, our previous studies have shown that host genetic background plays an important role in determining the sensitivity of the mice to insulin resistance, body weight gain, development of polyps in intestines and periodontitis, and other infectious diseases.^8^, ^21^, ^22^, ^23^, ^24^, ^28^, ^29^, ^30^ In the present study, the results confirm that the host genetic background is an important factor for defining the severity of the multimorbidity of the assessed and presented phenotypes.

In order to investigate the link between intestinal cancer, obesity and T2D, here we presented a comprehensive picture of the susceptibility status and profiles of two CC lines, IL557 and IL711, to diet and infection challenges, separately and together.^10^, ^22^, ^23^, ^24^ The heatmap analysis emphasizes the strong linear profiles between these phenotypes, especially between the BW and AUC results. The findings demonstrate the diversity between different strains under the same environmental conditions, and how the patterns and levels of the development of more than one disease can be studied at the same time in each strain. This multimorbidity analysis approach will help us to better understand the mechanism of each disease separately and how it is related to other diseases, under the same conditions. Identifying the gene(s) underlying these patterns will help us to better predict the development of many diseases, which will offer a new set of opportunities to prevent the early stages of each individual disease and its associated diseases.

In this study, we used a chow diet (CHD), which provides of 11% Kcal from fat, 24% from protein, and 65% from carbohydrates, and a high‐fat diet (HFD), which provides 42.0% Kcal from fat, 15.3% from protein, and 42.7% from carbohydrates. The major difference in Kcal content between CHD and HFD is the fat component, which appears to be 11% and 42%, for CHD and HFD, respectively, with overall difference of 31%. The difference in protein content is moderate, while there is a significant reduction in carbohydrate content in the HFD. Therefore, our results show that the T2D development is due to the significantly increased fat content in the HFD.

A number of previous studies have shown that the fibre content of a diet has a major effect on the gut microbiome.^31^, ^32^ In our study, the chow diet (Altromin 1324) has a crude fibre content, which is missing from the high fat diet (TD.88137). Since the fibre content affects the gut microbiome, which subsequently effects on the metabolism and immune function, it is therefore likely that the high‐fat diet will negatively affect the metabolism and immune function in the mice, because of its high fat‐content, which subsequently results in development of T2D.

Previous studies have shown that host genetic background is a crucial factor for the development of multiple polyps in the intestines, while the larger number of polyps, the higher chance that the tissue will develop cancerous cells.^33^ Using the CC model and the power of its huge genetic diversity, our study focused on genetic factors, with the ultimate aim of being able to predict intestinal cancer disease in its early stages by identifying specific genes underlying the multimorbidity of intestinal cancer, obesity and T2D. Our results have demonstrated the differences in the response of the two studied CC lines, which are known to have different genetic backgrounds,^12^, ^14^, ^16^, ^17^, ^18^, ^19^, ^21^, ^34^ to dietary and infection challenges, and have suggested that different host genes lead to different phenotypes, which vary between both sexes.

Since HFD‐induced free fatty acids, which damage the intestines, we may hypothesis is that the cytotoxicity of the abundant HFD‐derived free fatty acids in the intestinal lumen impairs the intestinal immune system.^33^ Our results showed that a high number of polyps developed in HFD‐fed mice, but interestingly, this effect varied between mice with different genetic backgrounds. This proves the influence of the host genetic background on the development of these diseases, whether considered separately, or as co‐ or multimorbidities. A recent study indicated that maintaining mice on HFD induced shortening of the small intestine and colon, and thinning of the ileum, which was defined as intestinal lipotoxicity. In metabolic syndromes, lipotoxicity usually indicates cytotoxicity to pancreatic islet β‐cells, causing abnormal glucose tolerance.^33^ In our study, the CC lines exhibited tremendous variation in glucose sensibility, body weight gain and polyp counts. We believe that by using this genetic reference population and expanding the number of the lines studied to 30 or more and using genome‐wide association studies (GWAS), we will be able to identify modifier genes associated with the multimorbidity of *these diseases*, as was successfully achieved in our previous studies.^12^, ^14^, ^15^, ^16^, ^17^, ^18^, ^19^, ^20^, ^22^, ^34^, ^35^, ^36^

In conclusion, it is believed that with providing more data on different CC lines, will promise to elucidate the components of the host genetic background that are involved in resistance to and rate of development of the multimorbidities of intestinal cancer, T2D and obesity induced by high fat and oral infection. Once obtained, such data can be used to predict the individual genetic risk factors underlying the development of these diseases and provide a platform for utilizing early prediction, prevention and treatment strategies.

## CONFLICT OF INTEREST

The authors have declared no conflicts of interest.

## AUTHOR CONTRIBUTIONS

Asal Milhem was involved in executing the project, data recording and analysis, and MS preparation. Hanifa J. Abu‐Toamih‐Atamni was involved in data analysis and MS preparation. Luna Karkar was involved in executing the project. Fuad A. Iraqi was involved in the project design, data analysis and MS preparation and approving its final version.
